# Comparative Evaluation of the Antitumor Effect of Rosmarinic Acid and Nordihydroguaiaretic Acid in Osteosarcoma Cells

**DOI:** 10.1021/acsomega.6c05639

**Published:** 2026-10-08

**Authors:** Flavia G. Iano, Adriano S. Pessôa, Mariana L. R. Sanches, Cíntia C. Tokuhara, Maiara S. B. Caires, Rodrigo C. Oliveira, Valdecir F. Ximenes

**Affiliations:** † Departamento de Química, Faculdade de Ciências, Universidade Estadual Paulista Júlio de Mesquita Filho (UNESP), Bauru, São Paulo 17033-360, Brazil; ‡ Departamento de Ciências Biológicas, Faculdade de Odontologia de Bauru, Universidade de São Paulo (FOB-USP), Bauru, São Paulo 17012-901, Brazil; § Center for Cardiovascular and Muscular Diseases, 4355 Sanford Burnham Prebys Medical Discovery Institute, La Jolla, California 92037, United States

## Abstract

Osteosarcoma is the most common type of bone cancer and is known for its high likelihood of metastasis. Rosmarinic acid (RA) and nordihydroguaiaretic acid (NDGA) are plant polyphenols that offer promising health benefits. From a molecular structure point of view, RA and NDGA are quite similar, but one difference stands out: hydrophobicity. Based on this feature, this work aimed to compare RA and NDGA in a panel of antioxidant assays and as cytotoxic and antiproliferative agents, inhibitors of cell migration, and inducers of intracellular reactive oxygen species (ROS) production in murine osteosarcoma cells (UMR-106) and preosteoblastic cells (MC3T3-E1). Our comparative study establishes that NDGA exerts markedly stronger cytotoxic, antiproliferative, and antimigratory effects than RA in osteosarcoma UMR-106 cells, associated with greater intracellular oxidative activity and disruption of redox homeostasis. While RA retains significant antioxidant activity and minimal cytotoxicity toward differentiated MC3T3-E1 osteoblastic cells, its comparatively low cytotoxicity in the present model suggests a more limited therapeutic effect than NDGA under the conditions tested. NDGA emerges as a potent experimental candidate for further investigation in osteosarcoma, although its translational potential remains limited by concerns regarding systemic toxicity and the need for validation in human osteosarcoma models. Future studies should aim to optimize NDGA delivery and selectivity, elucidate the molecular pathways involved, and explore synergistic combinations with standard therapies. Ultimately, the divergent activities of RA and NDGA underscore the importance of structural and physicochemical determinants in dictating the therapeutic potential of plant polyphenols against osteosarcoma.

## Graphical abstract

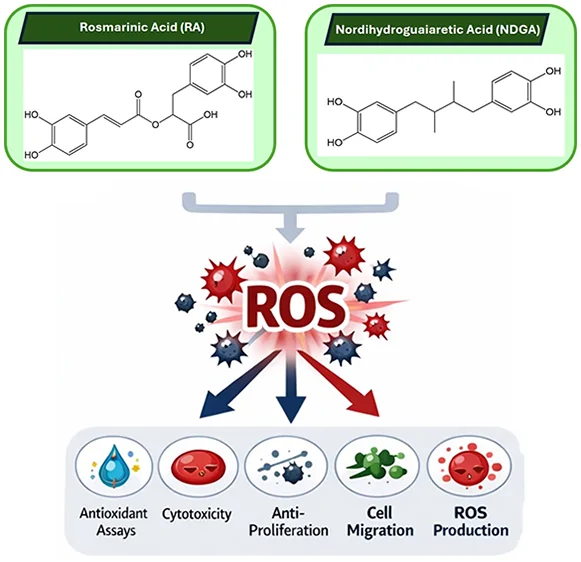

## 1 Introduction

Osteosarcoma (OS) is the most common type of bone cancer and is known for its high likelihood of metastasis. The incidence of OS is higher during adolescence, peaking at 8 to 11 million cases annually among 15–19 year-olds. The treatment protocol for osteosarcoma has not changed since the 1970s.^1,2^ Thus, many efforts have been made to identify promising alternatives for osteosarcoma treatment.^3–5^

Historically, thousands of herbs and their derivatives have been used in folk medicine to treat numerous diseases. From herbs, active principles are extracted, purified, and chemically characterized. Then, researchers initiate a new phase using purified compounds from natural sources in more sophisticated in vitro, cell-based, and in vivo studies. This is the case for rosmarinic acid (RA) and nordihydroguaiaretic acid (NDGA), which have more than 5000 papers listed in the PubMed database. RA is a potent polyphenol found in rosemary (*Rosmarinus officinalis*) extract. It has been recognized as an effective agent against various cancer cell lines and has exhibited an apoptotic effect on multiple types of cancer.^6–8^ Additionally, RA has been shown to enhance the apoptotic effect of the chemotherapy Adriamycin.^9,10^ NDGA is a natural lignin, primarily extracted from the creosote bush (*Larrea tridentata*), which is plentiful in the United States and Mexico. People in these regions have used the leaves in traditional medicine to treat diseases like rheumatism, arthritis, diabetes, pain, and inflammation.^11^ As expected, several carcinogenesis models have shown that NDGA can inhibit the growth of different human cancer types in both cell cultures and animal models.^6,12–14^

Tumor cells often face stress conditions and disrupted oxidative metabolism. In this context, reactive oxygen species (ROS) play a crucial role in regulating cell signaling and are involved in various stages of tumor progression.^15^ For instance, expression of NADPH oxidases (NOXs) and ROS production are important in the behavior of cancer cells.^16–18^ The interplay between ROS deregulation and tumor cells is one reason for the large number of studies investigating potential cancer treatments using natural polyphenols. However, the antioxidant properties of polyphenols are not the only chemical feature underlying the potential benefits of these phytochemicals. Another is their hydrophobicity, which directly influences access to membranes and the cell interior. As stated above, RA and NDGA are widely used as potential anticancer agents. From a molecular perspective, these compounds are very similar, but one difference stands out: hydrophobicity (Figure 1). Because this physicochemical property directly affects membrane diffusion and access to protein-binding sites, this work was designed to evaluate and compare their potency as cytotoxic agents against osteosarcoma cells. Given their redox similarity and significant differences in hydrophobicity, the comparison between RA and NDGA highlights the importance of each chemical property, as shown below.

**Figure 1:**
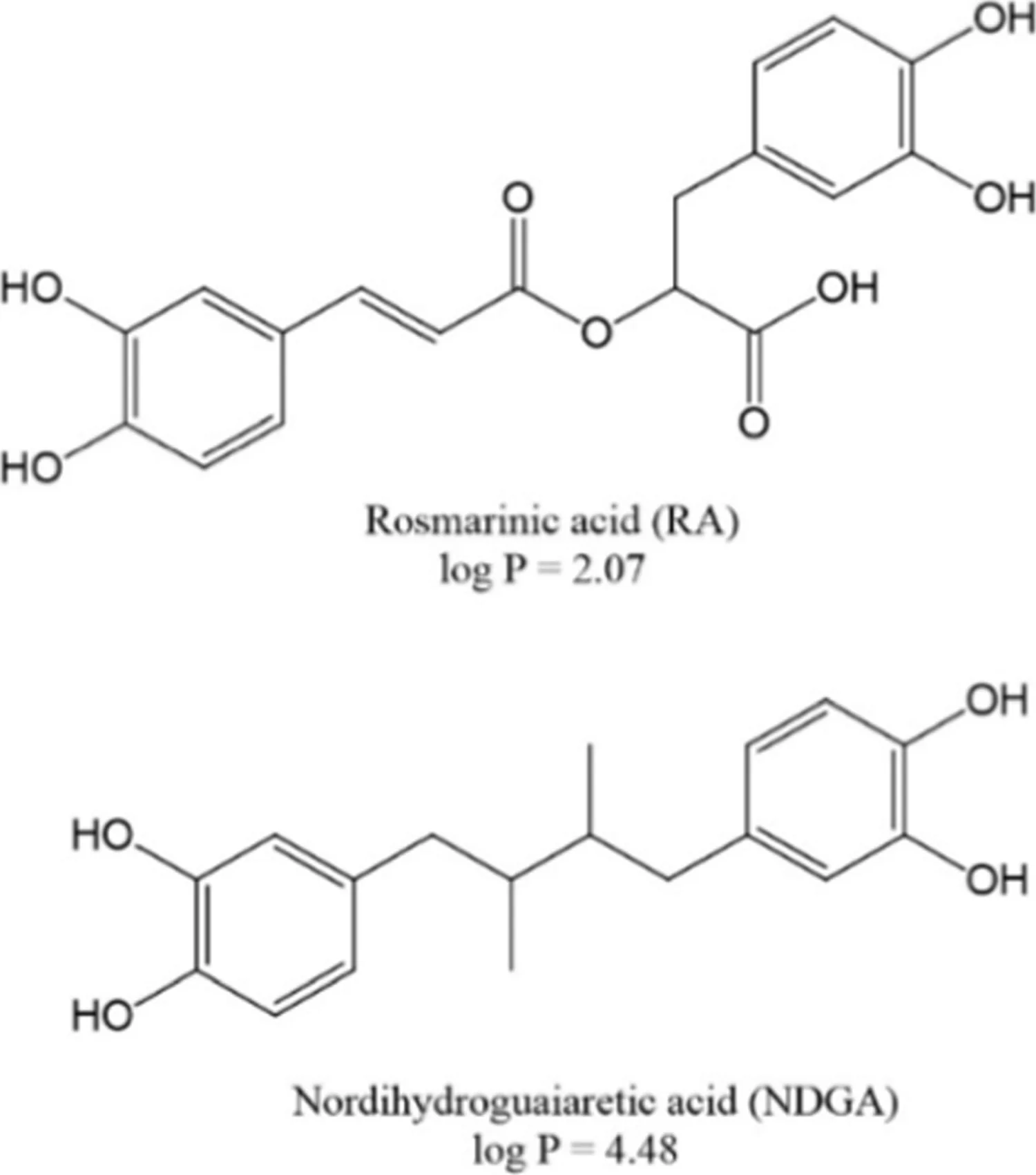
Molecular structures, hydrophobic index (log *P*) and acidity (p*K*_a_) of the compounds studied.

## 2 Results

### 2.1 RA Versus NDGA: Chemical Features

RA and NDGA are widely used bioactive compounds for treating various diseases, particularly as cytotoxic agents against tumor cells. From a molecular perspective, RA and NDGA are similar. The equal anodic peak potentials, RA (0.17 V) and NDGA (0.16 V), reflect the presence of catechol moieties in both molecules, which are oxidizable and responsible for redox properties.^19^ However, RA and NDGA differ significantly in hydrophobicity and acidity. As shown in Figure 1, the presence of a carboxylic group in RA, deprotonated in an aqueous medium (p*K*_a_ 3.57), makes it more hydrophilic, as evidenced by its higher water solubility compared to NDGA. RA is soluble in PBS (pH 7.2) at 15 mg/mL, while NDGA is soluble at 0.05 mg/mL.^20–22^ Instead, NDGA has an aliphatic carbon chain linking the catechol groups, which accounts for its hydrophobic nature. These structural features are reflected in their hydrophobicity (log *P*), which is significantly higher for NDGA (3.92) than for RA (0.86). These physicochemical differences are expected to affect the accessibility and diffusibility of molecules across the cell membrane and other organelles.

To compare the redox properties of RA and NDGA, we conducted three well-established antioxidant assays:^11^ (i) the DPPH scavenging assay, which measures the reduction of the DPPH free radical (Figure 2A); (ii) the FRAP assay, which evaluates the effectiveness of the tested compounds as reducing agents by converting ferric ions to ferrous ions (Figure 2B); (iii) the oxygen radical absorption capacity (ORAC) assay, which assesses the reactivity of potential antioxidants with peroxyl radicals (Figure 2C). As can be inferred, the protocols represent three very different chemical fundamentals. These include the reduction of a free nitrogen-centered radical (DPPH), the reduction of a metal ion (FRAP), and the reduction of an oxygen-centered radical (ORAC). The results displayed in Figure 2 show the typical analytical profile obtained in these assays, and Table 1 shows the values of the antioxidant parameters. As can be seen, both compounds have proven to be powerful antioxidants; however, no significant differences were observed between them.

**Figure 2:**
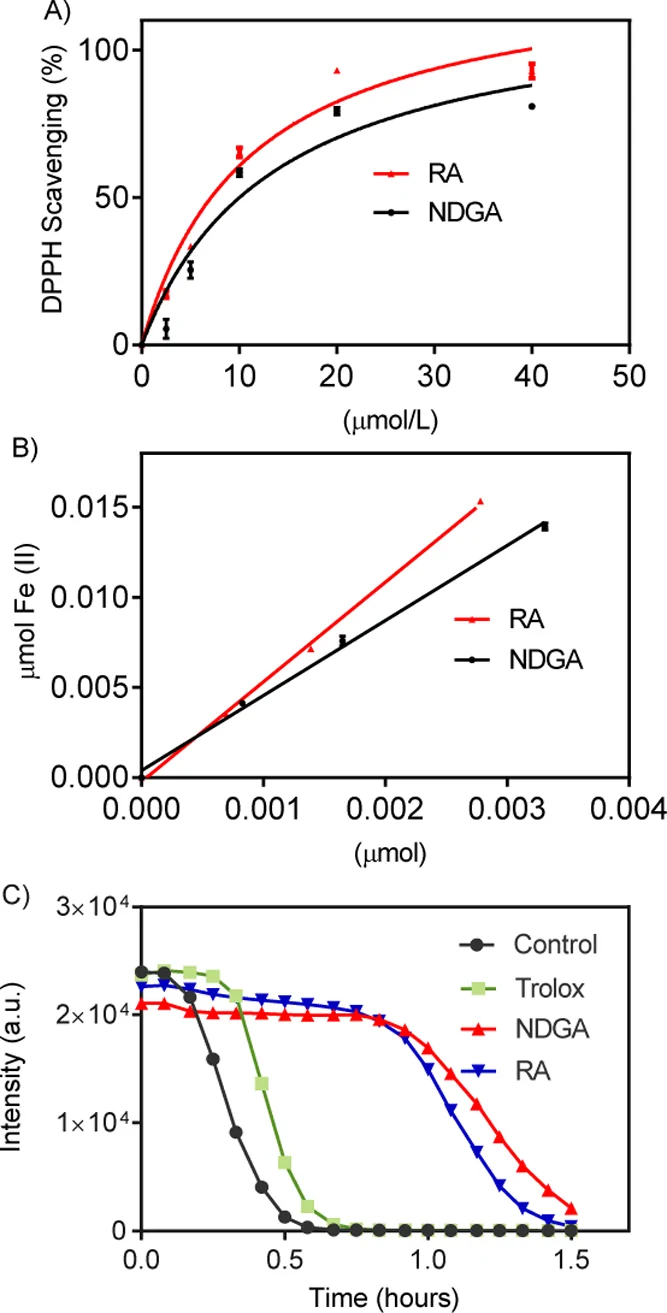
Free radical scavenging activity of RA and NDGA. (A) DPPH scavenging assay, (B) FRAP assay, and (C) ORAC assay.

**Table 1:** Rosmarinic Acid Versus Nordihydroguaiaretic Acid: Free Radical Scavenging Activity

	^a^DPPH (EC_50_)	^b^ORAC	^c^FRAP
RA	131.7 ± 9.5	5.50 ± 0.40	5.25 ± 0.18
NDGA	117.2 ± 14.1	4.98 ± 0.59	4.59 ± 0.30

a EC_50_ in μmol/L.

b Equivalence in Trolox (μmol Trolox/μmol compound).

c Equivalence in Fe^2+^ (μmol Fe^2+^/μmol compound). Results are represented as means and standard deviations of triplicates.

### 2.2 RA Versus NDGA: Cytotoxic Properties

After evaluating similarities in redox properties and detecting differences in hydrophobicity, the next step was to assess cytotoxic effects on normal and tumor cells. The chosen lineages were the normal MC3T3-E1 osteoblastic cells and UMR-106, a clonal derivative of transplantable rat osteosarcoma. The cytotoxic potential was evaluated using the MTT reduction assay, which measures mitochondrial activity.^23,24^ Figure 3 shows the concentration-dependent effect of both compounds on cell viability after 24 to 72 h of incubation. As shown, the difference between RA and NDGA was evident. While RA did not induce a statistically significant cytotoxic effect in MC3T3-E1 cells (Figure 3A,C,E), NDGA led to an almost complete loss of cell viability at 72 h of treatment (Figure 3B,D,F).

**Figure 3:**
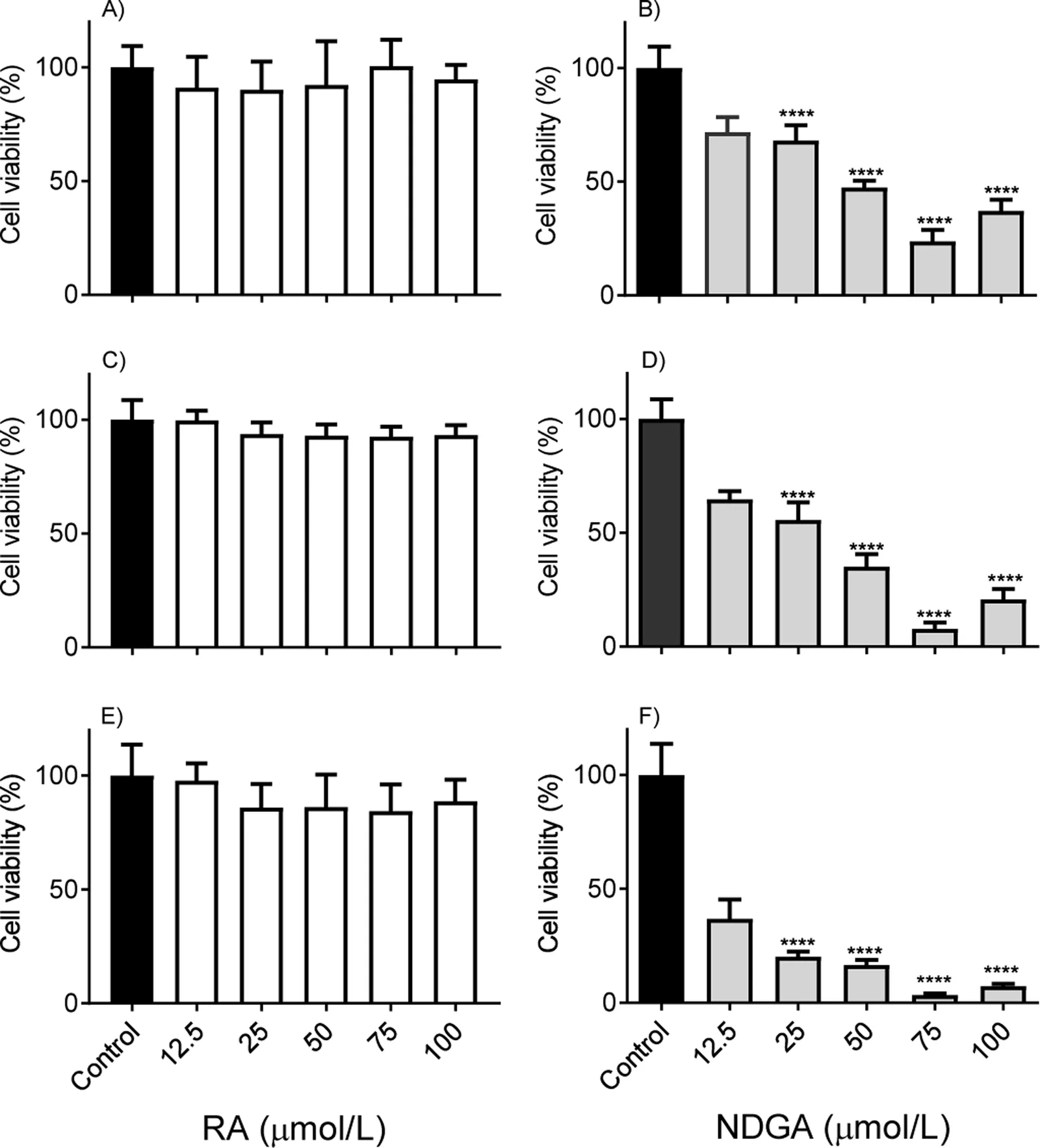
Rosmarinic (RA) versus nordihydroguaiaretic (NDGA) acids: cytotoxicity on osteoblasts (MC3T3-E1) evaluated by the MTT assay. Cell viability was evaluated after 24 h (A,B), 48 h (C,D), and 72 h (E,F) of treatment. Values are expressed as the mean ± standard deviation (%) from three independent assays. ****P < 0.0001 represents statistical significance compared to the control.

Following the same protocol, we evaluated and compared the cytotoxic effect on UMR-106 tumor cells (Figure 4). As shown, NDGA again exhibited a stronger cytotoxic effect than RA. It was also evident that UMR-106 cells were more susceptible to RA than the normal osteoblast MC3T3-E1.

**Figure 4:**
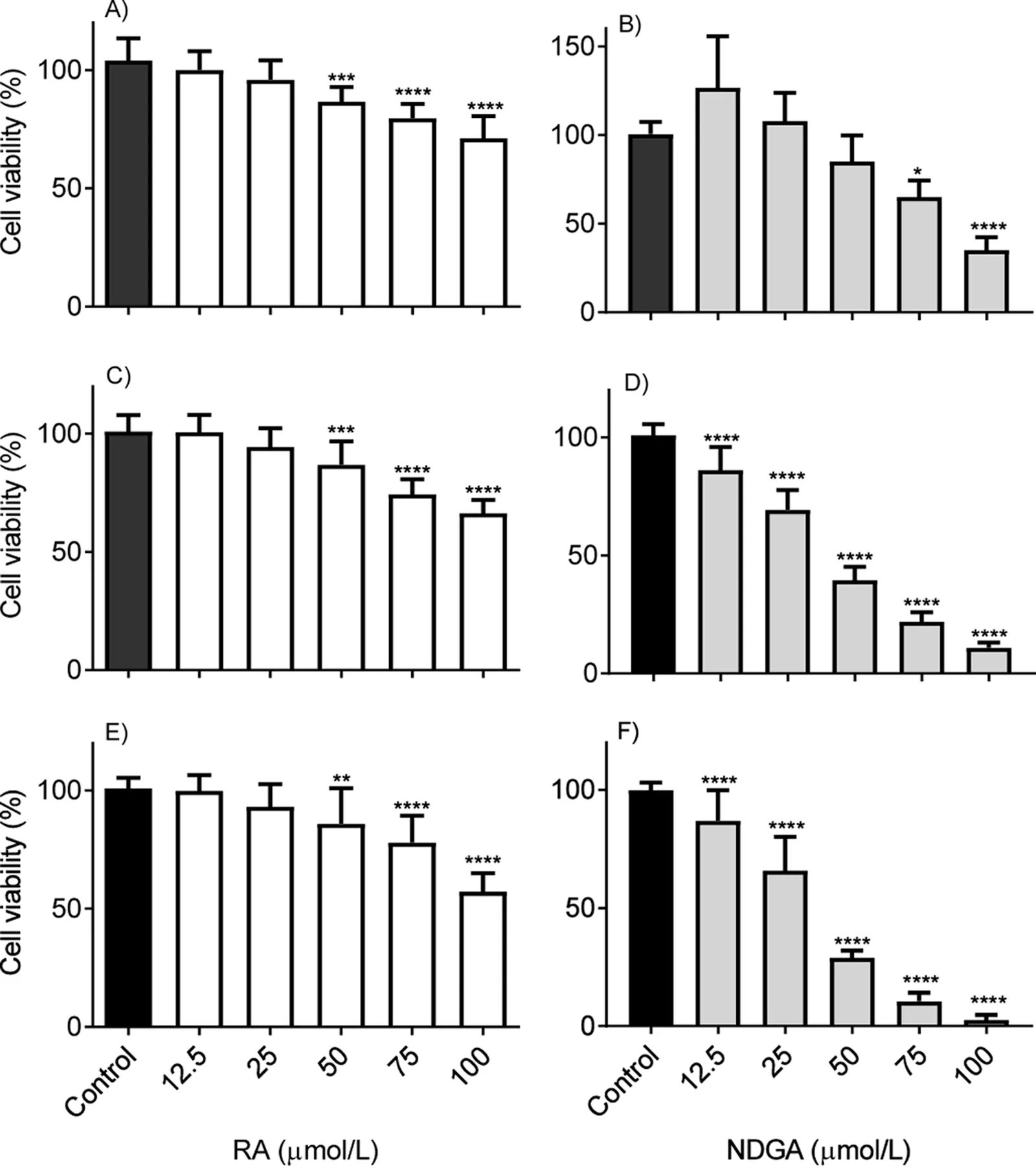
Rosmarinic (RA) versus nordihydroguaiaretic (NDGA) acids: cytotoxicity on osteosarcoma cells (UMR-106) evaluated by the MTT assay. The cell viability was evaluated after 24 h (A,B), 48 h (C,D) and 72 h (E,F) after treatment. Values are expressed as the mean ± standard deviation (%) from three independent assays. *p < 0.05, **p < 0.01, ***p < 0.001; ****p < 0.0001 represents statistical significance compared to the control.

Figure 5 shows the higher cytotoxic effect of NDGA compared to RA, with cell viability assessed at 48 h. The results demonstrate that, regardless of the concentration, NDGA was statistically significantly more effective than RA (Figure 5A). Based on this data, the cell viability ratio (RA/NDGA) was calculated and is shown in Figure 5B. As observed, a concentration-dependent exponential increase was noted, indicating that NDGA has a greater cytotoxic potency than RA on UMR-106 tumor cells.

**Figure 5:**
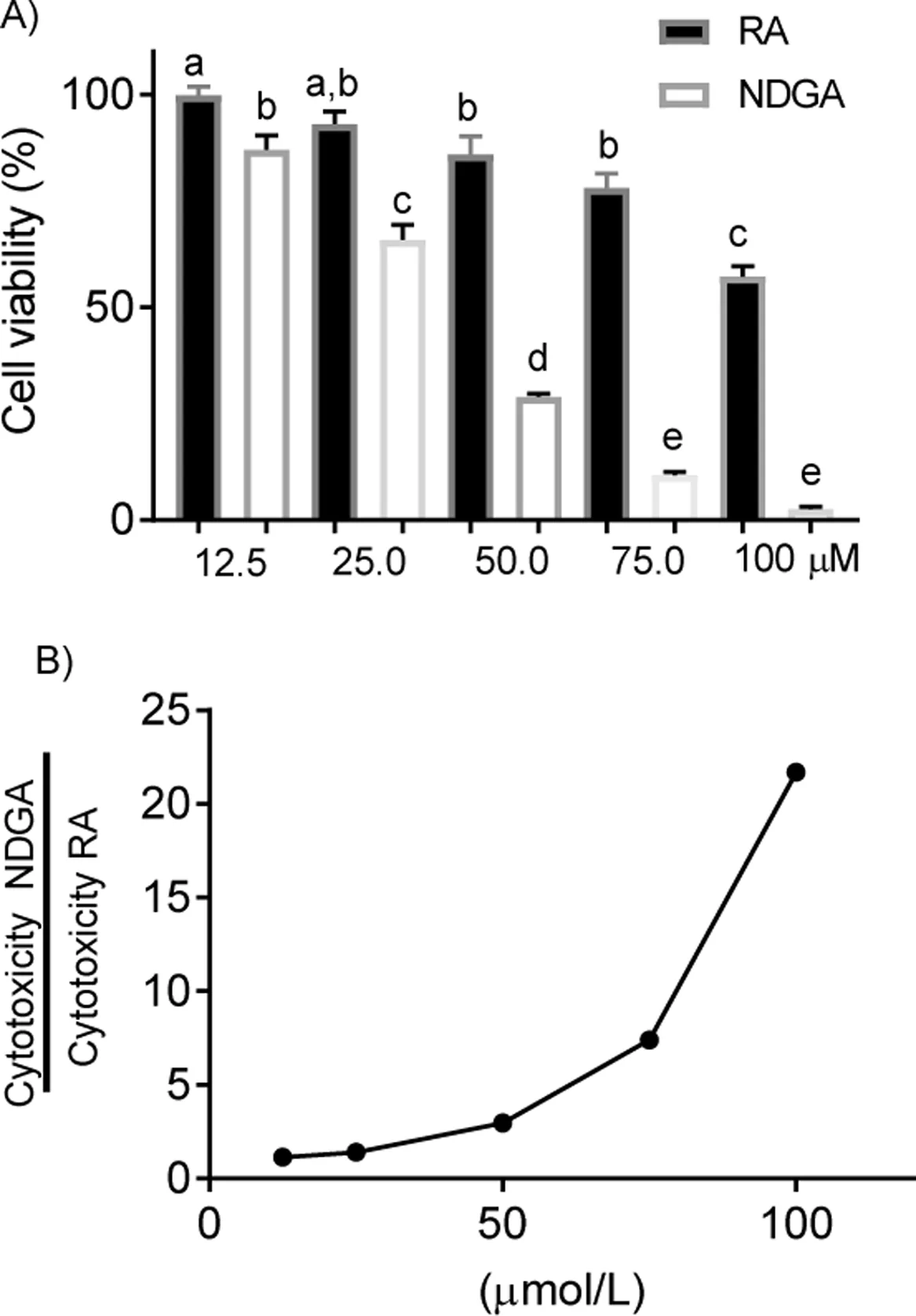
Comparative cytotoxicity: rosmarinic (RA) versus nordihydroguaiaretic (NDGA) on osteosarcoma cells (UMR-106) evaluated by the MTT assay after 48 h of treatment. (A) Comparison of concentrations: distinct letters denote statistical significance *p* < 0.05. (B) Cytotoxicity ratio measured by division of the average cell viability at each concentration.

To validate the previous results, the cytotoxicity of the compounds was compared for DNA damage using the crystal violet assay.^25,26^ The concentration-dependent responses are shown in Figure 6, and, consistent with the MTT assay, NDGA was more effective than RA.

**Figure 6:**
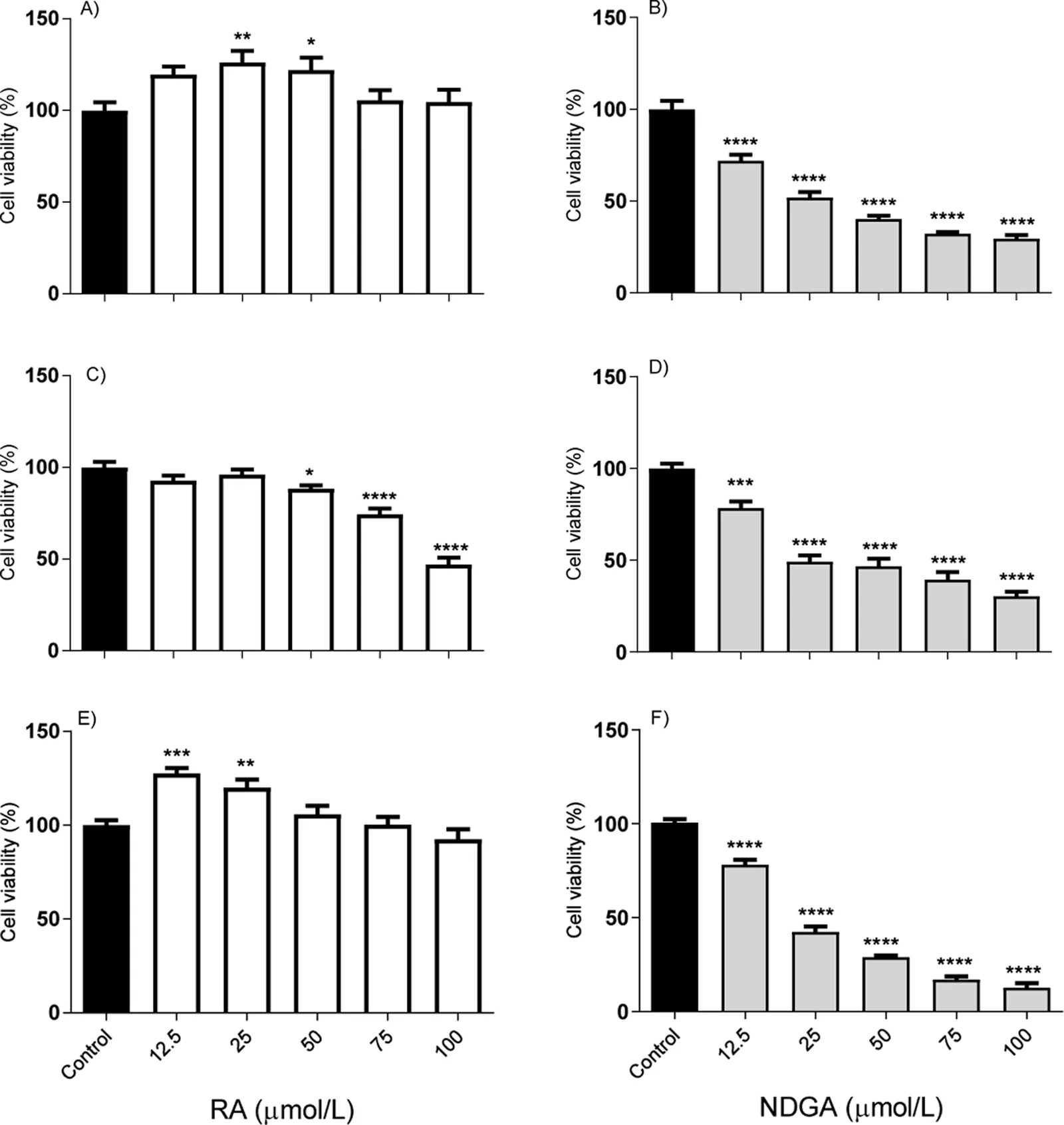
Rosmarinic (RA) versus nordihydroguaiaretic (NDGA) acids: cytotoxicity on osteosarcoma cells (UMR-106) evaluated by the crystal violet assay. The cell viability was evaluated after 24 h (A,B), 48 h (C,D) and 72 h (E,F) after treatment. Values are expressed as the mean ± standard deviation (%) from three independent assays. *p < 0.05, **p < 0.01, ***p < 0.001; ****p < 0.0001 represents statistical significance compared to the control.

### 2.3 RA Versus NDGA: Cell Migration Inhibition

NDGA demonstrated greater effectiveness as a potential drug against tumor cells^27^ in a cell migration assay using the wound-healing method. In this technique, a wound (slit) was made in a UMR-106 monolayer, and healing (cell migration, slit closure) was monitored. Cells were treated with compounds and compared with the control. Closure was evaluated at 24, 48, and 72 h. The NDGA IC_50_ concentration obtained in the MTT cell viability assay was used. Cell migration began at 24 h for all groups (Figure 7). In the control, the slit was almost completely closed at 48 h. As expected from the previous results, drug incubation delayed closure. In line with the cytotoxic assays, NDGA emerged as the most effective inhibitor. The difference between NDGA and RA was statistically significant at the 72 h^11^ (Figure 8).

**Figure 7:**
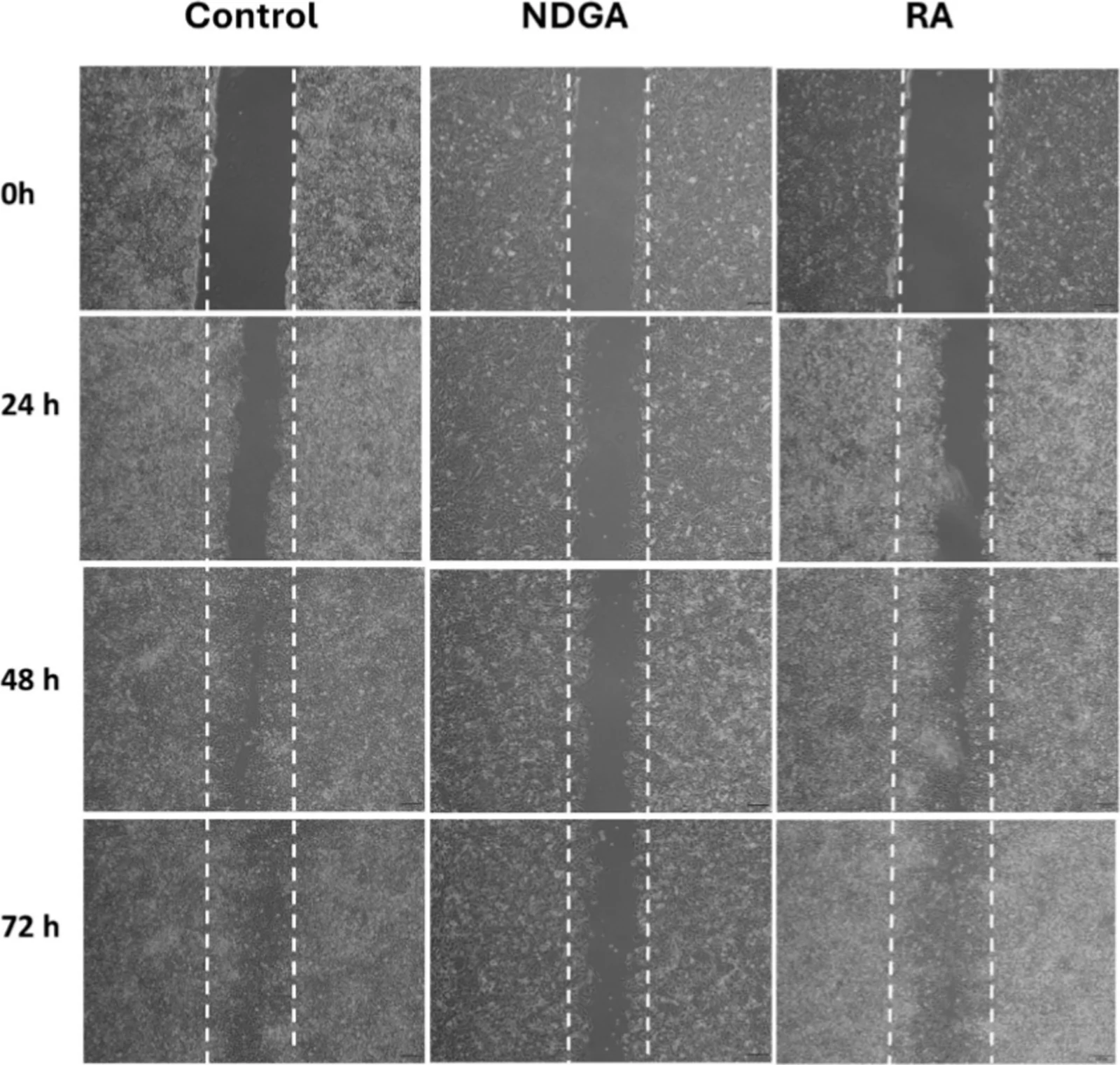
UMR-106 migration assay: wound-healing method observed at 0, 24, 48, and 72 h of treatment. NDGA and RA were used at 38 μM.

**Figure 8:**
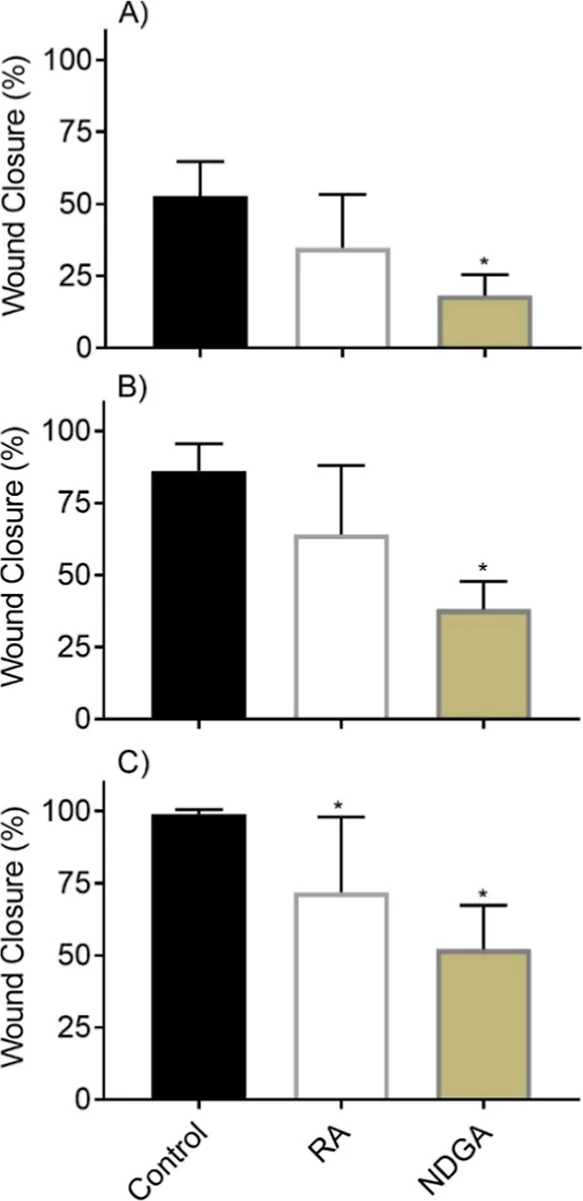
Inhibition of osteosarcoma cell migration was monitored by tracking the closure of a cell monolayer over (A) 24, (B) 48, and (C) 72 h. The concentration of RA and NDGA was 38 μM. Values are expressed as the mean ± standard deviation (%) from six independent assays. * Represents statistical significance compared to the control (*p* < 0.05).

### 2.4 RA Versus NDGA: Morphological Alterations

We performed qualitative morphological analysis by staining the cells with hematoxylin–eosin after 48 h of compound exposure.^28^ As shown, the control group had a higher cell number and well-defined cytoplasm. Both compounds caused cytoplasmic damage, reduced cell number, and reduced staining. Again, the damage caused by NDGA was qualitatively greater than that caused by RA (Figure 9A–C). Although the quantitative analyses of the nuclear and cytoplasmic areas (Figure 9D) did not reveal statistically significant differences, they supported the morphological changes observed following treatment with NDGA and RA.

**Figure 9:**
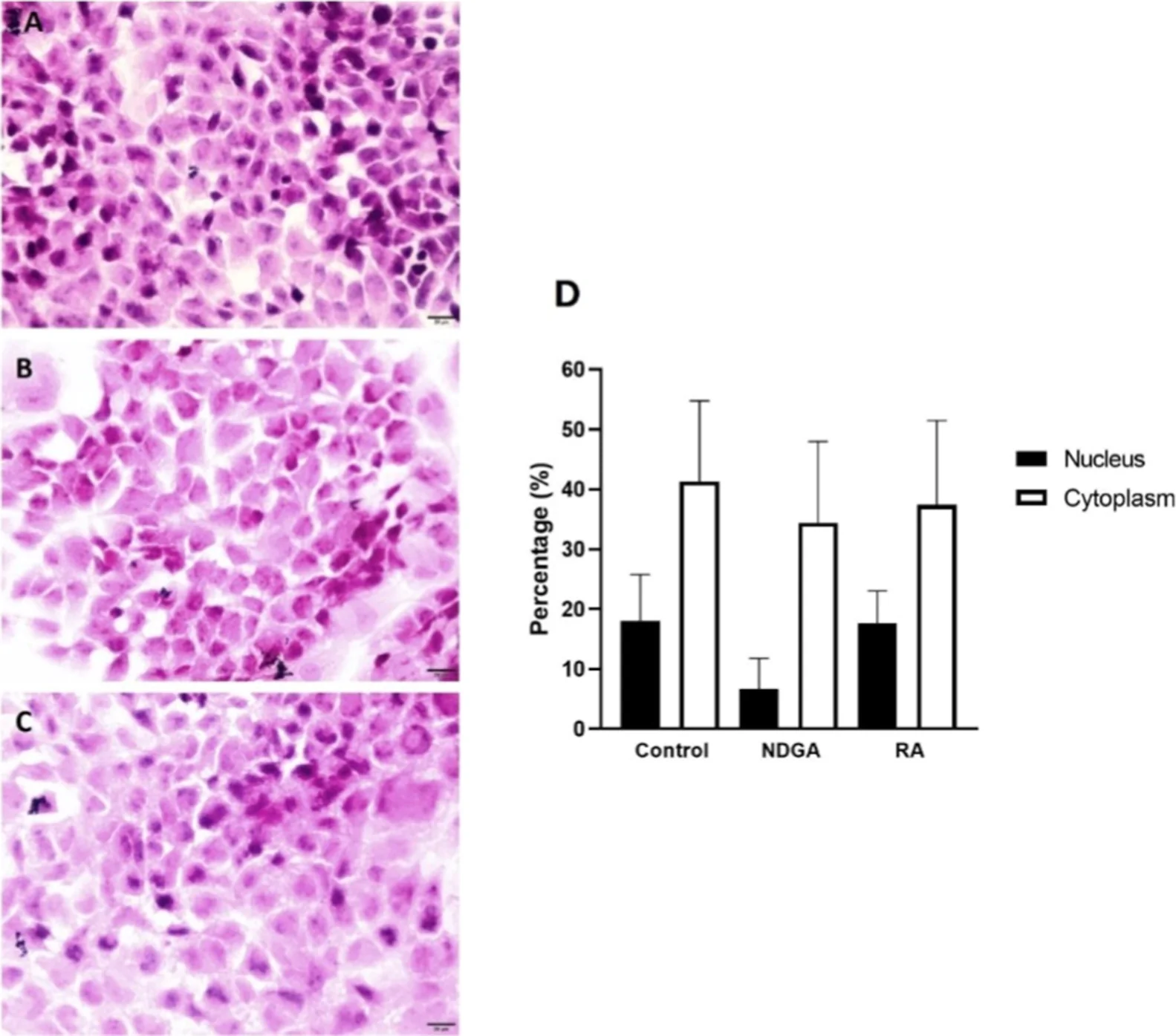
Morphological alteration induced by NDGA and RA in UMR-106: hematoxylin eosin staining. (A) Control cells, (B) NDGA, (C) RA. Representative images were captured using the Leica digital camera (Wetzlar, Germany) microscope at 40× magnification. (D) Analysis of nuclear and cytoplasmic areas using Fiji/ImageJ for quantification.

### 2.5 RA Versus NDGA: Cell Proliferation

The cytotoxic effect was also evaluated by flow cytometry using the CFSE:7-AAD cell proliferation assay. As shown, at the same concentration of RA and NDGA, only NDGA altered the UMR-106 features. NDGA treatment caused a significant decrease in the peak, peak splitting, and a rightward shift, indicating fewer cell divisions (Figure 10).

**Figure 10:**
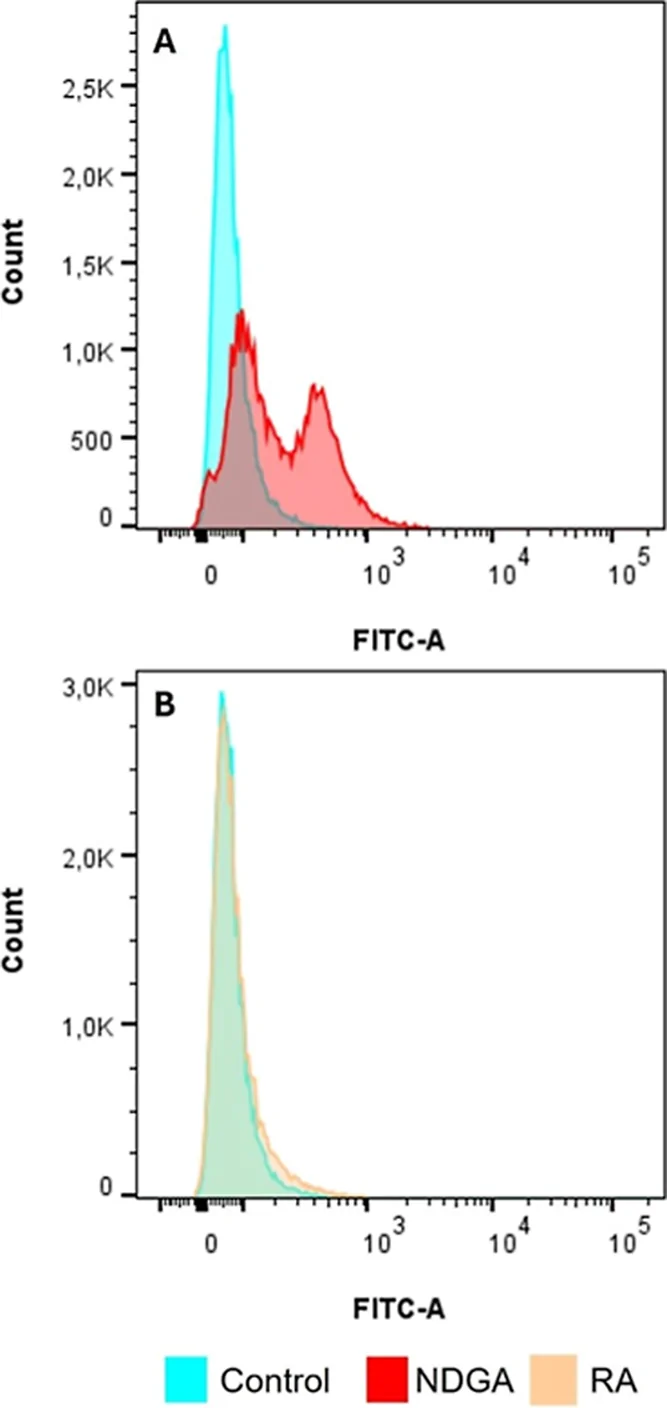
Proliferation analysis. Flow cytometry analysis of UMR-106 cells with CSFE staining. (A) Control cells and NDGA, (B) control cells and RA.

### 2.6 RA Versus NDGA: Generating Intracellular ROS

Given the cytotoxic effects of the compounds, their redox-active properties, and the well-established susceptibility of tumor cells to ROS, we next evaluated intracellular oxidant generation. We measured ROS levels using the DCFH-DA assay. The results in Figure 11 confirmed our hypothesis, as intracellular ROS levels were higher in cells treated with RA and NDGA than in untreated control. Moreover, NDGA produced statistically significantly higher levels, consistent with its greater cytotoxic effect. This increased oxidative response corresponded with the enhanced cytotoxic effect of NDGA observed in the viability assays, indicating a link between intracellular oxidative stress and the differing cytotoxic activity of the two compounds.

**Figure 11:**
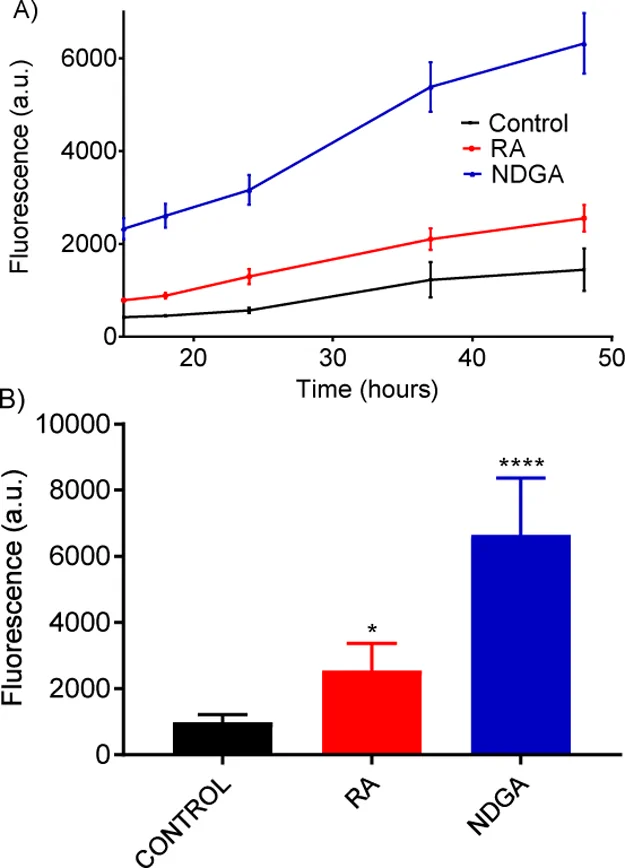
Intracellular production of ROS. (A) Time-dependent and (B) total production of ROS. The UMR-106 were loaded with 2′,7′-dichlorofluorescein diacetate (DCFH-DA), then exposed to RA and NDGA for 48 h. Values are expressed as the mean ± standard deviation (%) from six independent assays. *p < 0.05 and ****p < 0.0001 represent statistical significance compared to the control.

### 2.7 RA Versus NDGA: Apoptosis and Necrosis

Cells were stained with Annexin V-FITC and DRAQ7 to evaluate live cells, early apoptosis, late apoptosis, and necrosis by examining differences in plasma membrane integrity and permeability. Flow cytometry analysis (A) and subsequent statistical analysis (B) were performed on osteosarcoma cells (UMR-106) treated with NDGA and RA over a 48 h period (Figure 12).

**Figure 12:**
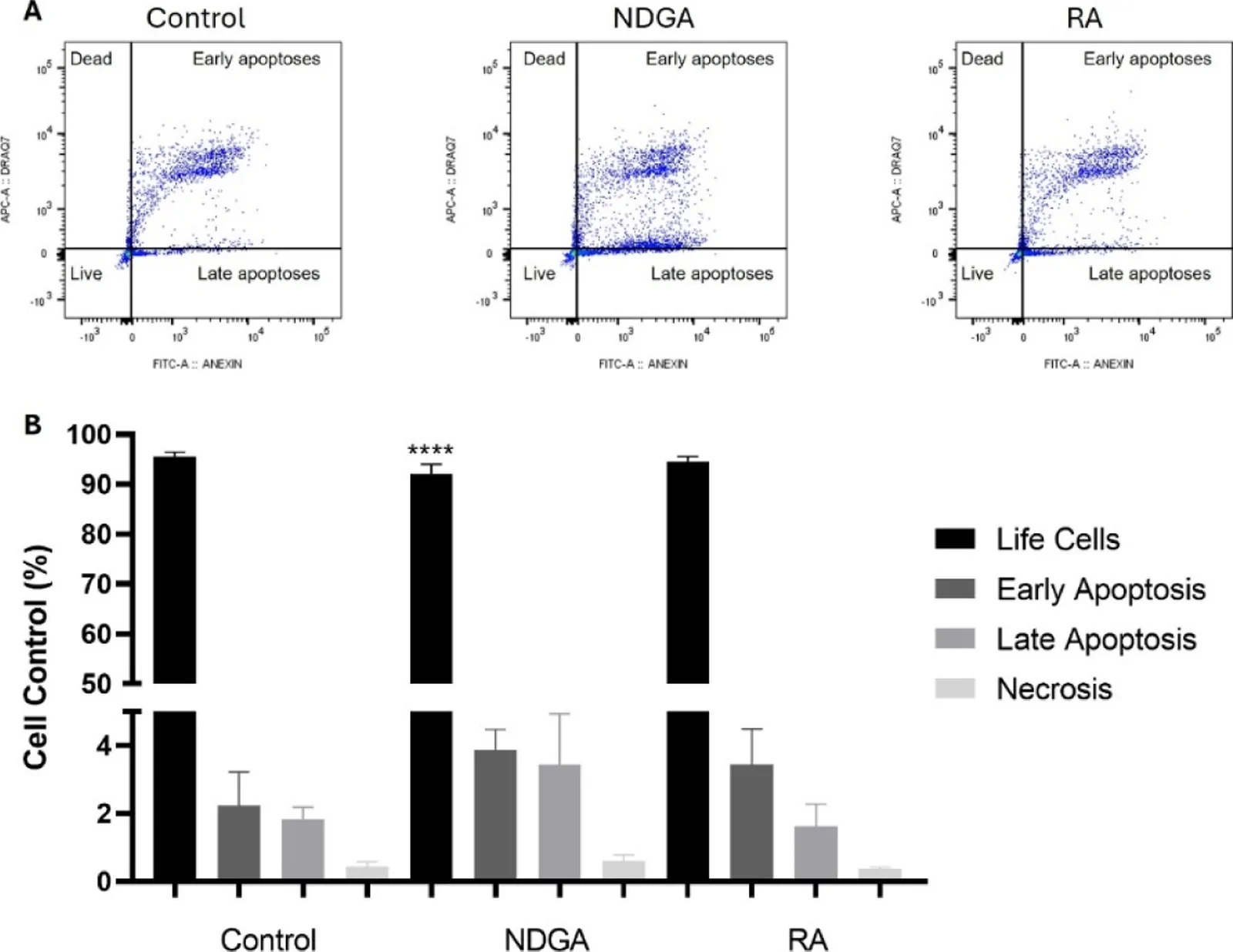
Flow cytometry analysis (A) and statistical analysis (B) of apoptosis and necrosis provoked by NDGA and RA. Flow cytometry analysis (A) and statistical analysis (B) of osteosarcoma cells (UMR-106) treated with NDGA and RA within 48 h. Values expressed as means ± standard deviation. *p < 0.0001 represents statistical differences when compared to the control group.

## 3 Discussion

Osteosarcoma remains a major therapeutic challenge because of its aggressive behavior, metastatic potential, and the limited treatment options for patients with advanced or recurrent disease.^29,30^ In this context, natural products and polyphenolic compounds have attracted considerable interest in their structural diversity and their ability to modulate cellular redox status, proliferation, and survival pathways.^30–32^ The present study compared two structurally related polyphenols, rosmarinic acid (RA) and nordihydroguaiaretic acid (NDGA), in rat osteosarcoma UMR-106 cells and differentiated mouse MC3T3-E1 osteoblastic cells. The central finding was that RA and NDGA showed similar antioxidant activity in cell-free assays but markedly different cellular effects. NDGA was substantially more cytotoxic and antiproliferative, produced a greater increase in intracellular ROS, and inhibited cell migration more strongly than RA. These differences provide evidence that chemical antioxidant capacity alone does not predict the biological activity of structurally related polyphenols.

RA and NDGA share catechol moieties, which are important contributors to their redox activity because they can oxidize and donate hydrogen atoms or electrons to reactive species.^19,33–35^ Consistent with this structural similarity, the present study found no significant difference between RA and NDGA in DPPH, FRAP, or ORAC assays. These assays, however, assess chemical reactivity under cell-free conditions and therefore do not replicate the physicochemical and biological processes that govern cellular activity. This distinction is particularly relevant to the present comparison because RA and NDGA differ markedly in hydrophilicity, solubility, and lipophilicity. RA contains a carboxylic acid group and has a reported log *P* of approximately 0.86, whereas NDGA contains an aliphatic chain linking its catechol groups and has a considerably higher log *P* of approximately 3.92.^19,36^ Such differences may affect membrane interactions, intracellular accessibility, and distribution, providing a plausible explanation for their divergent cellular effects. However, because intracellular uptake and subcellular accumulation were not directly measured, lipophilicity should be considered a potential contributing factor rather than a demonstrated mechanism.

The cytotoxicity results showed a distinct difference in the biological activities of the two compounds. RA had limited effects on MC3T3-E1 cells^37^ and a relatively weaker effect on UMR-106 cells. In contrast, NDGA resulted in a concentration- and time-dependent decrease in cell viability in both models, with a more significant response observed in osteosarcoma cells.^38^ The greater sensitivity of UMR-106 cells to NDGA is consistent with the concept that malignant cells may be particularly vulnerable to additional disturbances in redox homeostasis because they frequently operate under increased basal oxidative stress and depend on antioxidant systems to maintain survival.^39–42^ The intracellular ROS results are particularly informative when considered alongside the cell-free antioxidant assays. Both RA and NDGA increased DCFH-DA fluorescence in UMR-106 cells, whereas NDGA produced a significantly greater increase. Thus, although both compounds efficiently scavenged or reduced oxidants in chemical assays, their effects within cells were associated with an altered oxidative state. This apparent discrepancy is biologically plausible because the intracellular response to a polyphenol depends not only on its intrinsic radical-scavenging capacity but also on cellular uptake, metabolism, redox cycling, interactions with membranes and proteins, and the capacity of endogenous antioxidant systems. In this context, NDGA may exert a net pro-oxidant cellular effect despite its strong chemical antioxidant activity. Importantly, however, the DCFH-DA assay used here does not identify the specific ROS involved or establish their intracellular source. Therefore, the present results demonstrate increased intracellular oxidative activity, rather than a specific ROS-generating mechanism.

The association between increased oxidative activity and NDGA-induced cytotoxicity is further supported by the complementary cellular assays. NDGA produced greater morphological alterations, reduced cell number and cytoplasmic integrity, decreased proliferation, and greater changes in the proportions of viable, apoptotic, and necrotic cells than RA. These findings indicate that the reduction in viability was accompanied by broader alterations in cell survival and proliferation rather than representing an isolated metabolic effect. Nevertheless, the present experiments do not establish that ROS accumulation is causally responsible for NDGA-induced cell death. Previous studies provide a possible mechanistic framework: NDGA has been reported to promote glutathione oxidation, lipid peroxidation, mitochondrial depolarization, and apoptosis in other cellular systems, including a model lacking detectable lipoxygenase activity.^43^ This evidence is particularly relevant because it indicates that NDGA-induced cell death may involve disruption of redox and mitochondrial homeostasis independently of a simple lipoxygenase-inhibition mechanism. However, mitochondrial membrane potential, glutathione levels, caspase activation, and ROS source were not measured in the present UMR-106 model and should therefore be investigated in future studies.

The antimigratory effect of NDGA provides an additional functional distinction between the compounds. NDGA inhibited wound closure more strongly than RA at the concentration evaluated. Because cells were exposed to mitomycin C before the scratch was generated, the contribution of cell proliferation to wound closure was minimized, supporting interpretation of this result as an effect on collective cell migration. Nevertheless, wound-healing assays measure two-dimensional collective movement and cannot by themselves establish an antimetastatic effect. Therefore, the present results support an antimigratory effect of NDGA in UMR-106 cells, but not a conclusion that NDGA inhibits osteosarcoma metastasis. This distinction is important because migration and invasion involve coordinated regulation of cytoskeletal dynamics, adhesion, extracellular matrix interactions, and signaling pathways.^44^ Previous studies have reported antimigratory activity of NDGA in other cancer models, including effects associated with lipoxygenase-related pathways,^6,13,45,46^ providing biological context for the present observation.

Comparison with RA is also important for interpreting the translational significance of these findings. The relatively low cytotoxicity of RA toward MC3T3-E1 cells, together with its strong chemical antioxidant activity, distinguishes it from NDGA and suggests that the two compounds may have fundamentally different biological profiles. Importantly, the weaker effect of RA in UMR-106 cells should not be interpreted as evidence that RA lacks antitumor activity.^38^ RA has been shown to inhibit the proliferation, migration, and invasion of human osteosarcoma U2OS and MG-63 cells, while also inducing apoptosis through the DJ-1/PTEN/PI3K/Akt signaling pathway.^47,48^ Thus, the comparatively modest effect observed here may reflect differences in species, cellular phenotype, concentration, exposure conditions, or molecular context rather than an intrinsic absence of antitumor activity.

Conversely, the greater potency of NDGA must be considered together with its known toxicity. NDGA has been associated with hepatotoxicity and nephrotoxicity, limiting its straightforward development as a systemic therapeutic agent.^49,50^ In mice, intraperitoneal administration of NDGA was associated with an LD_50_ of approximately 75 mg/kg, together with clinical signs and hepatic injury.^50^ Converting the higher concentration used here (100 μM) yields approximately 30 mg/kg, which is low compared with the reported LD_50_ in mice. The results suggest that the concentration used in this cellular study is likely safe. However, cell-based experiments do not account for factors such as absorption, protein binding, metabolism, tissue distribution, clearance, or overall systemic exposure. Therefore, while this study demonstrates NDGA potency at the cellular level, it cannot confirm whether these effective concentrations can be achieved in vivo without unacceptable toxicity.

Translational interpretation is constrained by the cellular models utilized. UMR-106 is a rat osteosarcoma cell line, while MC3T3-E1 is a mouse preosteoblastic cell line.^37^ Differentiation of MC3T3-E1 cells was used to obtain a more differentiated osteoblastic phenotype for comparison, consistent with the established use of this model in studies of osteoblast development.^51^ Nevertheless, neither model reproduces the molecular heterogeneity of human osteosarcoma. Differences in species, genetic background, oncogenic alterations, metabolism, and drug response may influence the effects of RA and NDGA. The absence of human osteosarcoma models is therefore an important limitation and should be explicitly acknowledged. Future studies should evaluate these compounds in multiple human osteosarcoma cell lines and, preferably, in three-dimensional spheroids, organoid models, or patient-derived systems before conclusions regarding translational efficacy are drawn.

Finally, the present findings identify several specific experimental approaches required to determine whether the association between NDGA, oxidative activity, and cytotoxicity is mechanistically causal. ROS scavenging or antioxidant rescue experiments could determine whether attenuation of oxidative activity reduces NDGA-induced cell death. Measurements of glutathione homeostasis, mitochondrial membrane potential, mitochondrial ROS, and caspase activation could further establish whether redox and mitochondrial dysfunction precede apoptosis. In addition, evaluation of NOX proteins or enzymatic activity would be more informative than mRNA expression alone for determining whether NADPH oxidases contribute to the observed oxidative phenotype. These approaches would directly address the mechanistic limitation identified in the present study and distinguish ROS-associated cytotoxicity from ROS-dependent cytotoxicity.

Overall, the present study demonstrates that two structurally related polyphenols with comparable chemical antioxidant capacity can display markedly different biological effects in osteosarcoma cells. NDGA showed greater cytotoxic, antiproliferative, antimigratory, and intracellular oxidative effects than RA, whereas RA displayed substantially lower cytotoxicity toward the osteoblastic model. The data support an association between the stronger cellular activity of NDGA and altered intracellular redox status, but they do not establish ROS as the direct cause of cell death or identify its molecular source. The greater lipophilicity of NDGA represents a plausible contributor to its divergent cellular activity, although this hypothesis requires direct assessment of cellular uptake and distribution. Taken together, NDGA emerges as a potent experimental compound for further investigation, but its systemic toxicity and the limitations of the current nonhuman models must be considered. Further mechanistic and translational studies are necessary to determine whether the differential activities of RA and NDGA can be exploited safely in human osteosarcoma.

## 4 Materials and Methods

### 4.1 Cell Culture

Rat osteosarcoma UMR-106 (CRL 1661) cells were cultured in Dulbecco’s modified Eagle medium (DMEM) from Sigma-Aldrich, while mouse preosteoblasts MC3T3-E1 Subclone 4 (CRL 2593), used as a control, were cultured in minimum essential medium Eagle, alpha modification (MEM α) from Gibco. Both cell lines were obtained from ATCC (Rockville, Maryland, USA). The culture medium was supplemented with 10% fetal bovine serum (FBS, Gibco) and 1% penicillin (10,000 IU/mL) and streptomycin (10,000 μg/mL) from Gibco. The cells were then placed in a CO_2_ incubator for the subsequent experiments.^26,52^

#### 4.1.1 Cell Differentiation

Differentiation was carried out to achieve a more mature osteoblastic phenotype, providing a more physiologically relevant nonmalignant comparator for the osteosarcoma model.^51^ MC3T3-E1 cells were differentiated using osteogenic medium, which consisted of αMEM supplemented with 10% FBS, 50 μg/mL ascorbic acid, and 10 mM β-glycerophosphate, and were cultured for 4 days.^51–53^

### 4.2 Rosmarinic Acid (RA) and Nordihydroguaiaretic Acid (NDGA)

The compounds were dissolved in ethanol, and a fresh stock solution of 100 mM was prepared before diluting it in complete medium for the experimental groups.

### 4.3 MTT Assay

Cell viability was assessed by measuring mitochondrial activity using the MTT assay^52^ (3-(4,5-dimethylthiazol-2-yl)-2,5-diphenyltetrazolium bromide) from Sigma-Aldrich. UMR-106 and MC3T3-E1 cells were plated at 5 × 10^3^ and 3 × 10^3^ cells per well, respectively. After 48 and 24 h of adhesion, the UMR-106 and MC3T3-E1 cells were differentiated in osteogenic medium, as described in Section 4.1.1. Following the adhesion period for UMR-106 and the differentiation period for MC3T3-E1, the cells were treated with NDGA and RA for 24, 48, and 72 h. Both compounds were initially tested at concentrations of 12.5, 25, 50, 75, and 100 μM. After the specified intervals, the treatments were removed, and 110 μL of MTT solution (0.5 mg/mL in culture medium) was added to each well. The plates were then incubated at 37 °C for 4 h. To solubilize the formazan crystals, DMSO was added to the wells, and after 30 min, absorbance was measured at 550 nm using a microplate reader (Synergy Mx monochromator multimode microplate reader, BioTek Instruments Inc., Winooski, Vermont, USA). Data analyses were performed using GraphPad Prism software.^23,24,52^

### 4.4 Violet Crystal Assay

The crystal violet assay was conducted using UMR-106 under the same plating conditions as the MTT assay. After 24, 48, and 72 h of treatment, the supernatant was removed and replaced with 50 μL of a 0.5% crystal violet solution. The mixture was then incubated at room temperature for 20 min. Following incubation, the plate was washed under running tap water until the stain was completely removed, and it was left at room temperature to dry for 16 h. After drying, 200 μL of methanol was added to each well, and the plate was incubated for another 20 min at room temperature on a microplate shaker to dissolve the crystals. Absorbance was measured at 570 nm using a Synergy Mx monochromator multimode microplate reader (BioTek Instruments Inc., Winooski, Vermont, USA).^26,52,54^

### 4.5 Hematoxylin–Eosin Assay

UMR-106 cells (5 × 10^4^) were plated on 13 mm round glass coverslips in a 24-well plate. After 48 h of cell adhesion, the culture medium was replaced with a solution containing 38 μM of NDGA and RA. The control group was maintained in complete medium. Following 48 h of treatment, the cells were fixed in 10% neutral-buffered formalin for 30 min and then washed with PBS. Next, a hematoxylin solution was applied for 3 min, followed by color separation using 0.5% alcohol acid. The cells were then counterstained with 1% eosin for 2 min, dehydrated through a gradient of ethanol, soaked in xylene, and mounted using Entellan mounting medium. Representative images were captured with a Leica microscope (Wetzlar, Germany) using a 40× magnification.^52,55^ The nuclear and cytoplasmic areas were quantified using Fiji/ImageJ software.

### 4.6 Apoptosis Assay

UMR-106 cells were plated at a density of 2 × 10^5^ cells per well in 6-well plates. After 48 h, the culture medium was replaced with the respective treatments: 38 μM NDGA, RA, or a control. Following another 48 h of treatment, the cells were passaged, washed twice with cold PBS, and centrifuged at 1200 rpm. Approximately 5 × 10^5^ cells were then resuspended in 250 μL of 1× Annexin V binding buffer and incubated with 5 μL of Annexin V FITC (Cat: 556419, BD Pharmingen) and 2.5 μL of DRAQ7 (Cat: 564904, BD Pharmingen) in the dark for 10 min at room temperature. The fluorescence intensity of the cells was analyzed using a BD FACSAria Fusion flow cytometer (BD Biosciences, San Jose, CA). Data were analyzed using FlowJo v10 software (FlowJo LCC, OR, USA), and cells were quantified as live (AV–/DRAQ7–, Q4), necrosis (AV–/DRAQ7+, Q1), early apoptosis (AV+/DRAQ7–, Q3), and late apoptosis (AV+/DRAQ7, Q2).^52,56^

### 4.7 Wound Healing Assay

UMR-106 cells were seeded at a density of 6 × 10^5^ cells per well in 6-well plates and incubated in a humidified environment at 37 °C with 5% CO_2_ for 48 h to promote adhesion and achieve confluence. Subsequently, the wells were treated with 5 μg/mL of mitomycin C (Sigma-Aldrich) for 2 h, washed with PBS, and then slit vertically using a 1000 μL pipet tip. The wells were washed three times with PBS and treated with NDGA and RA at 38 μM. The plates were imaged at 0, 24, 48, and 72 h after treatment using a microscope with phase-contrast optics, coupled with an Olympus U-TV0.5XC-3 digital camera equipped with a 4× objective. The percentage (%) of the crack area was calculated by ImageJ software.^26,52,57^

### 4.8 Reactive Oxygen Species Assay

UMR-106 cells were plated at a density of 2 × 10^4^ cells per well in black 96-well plates with a clear bottom and incubated at 37 °C under 5% CO_2_ for 24 h to promote adhesion. After incubation, the culture medium was removed, and the wells were washed with PBS. The wells were then incubated with 100 μL of 10 μM DCFH-DA in culture medium for 45 min at 37 °C under 5% CO_2_. Following this incubation, the DCFH-DA solution was replaced with treatments (38 μM NDGA and RA) and the control group (DMEM + 10% FBS), which were added to the culture without phenol red, maintaining 10% FBS. Fluorescence intensity was analyzed at 0, 24, and 48 h using a spectrophotometer with 495 nm excitation and 530 nm emission.^52,58^

### 4.9 DPPH Scavenging Assay

RA and NDGA (0–40 μM) were incubated for 30 min with 100 μM of 2,2-diphenyl-1-picrylhydrazyl (DPPH) in ethanol,^59^ kept in the dark. The scavenging activity was evaluated spectrophotometrically at 517 nm, using the absorbance of unreacted DPPH radical as the control. The scavenging activity was calculated as: [(absorbance of control – absorbance of sample)/(absorbance of control)] × 100.^60^

### 4.10 Ferric Reducing Antioxidant Power (FRAP) Assay

The reagent was prepared using the following components: 1 mL of 2,4,6-tris­(2-pyridyl)-*s*-triazine (TPTZ) at a concentration of 10 mM in 40 mM HCl, 1 mL of FeCl_3_ at a concentration of 20 mM in water, and 10 mL of 300 mM sodium acetate buffer (pH 3.6).^59^ Various concentrations of RA and NDGA (10 μL each) were incubated with 290 μL of the FRAP reagent for 30 min in the dark at room temperature. Absorbance was measured at 593 nm, using a mixture of 10 μL of 50 mM phosphate buffer (pH 7.0) and 290 μL of FRAP as the blank. The relative antioxidant efficacy was determined from the slope of the analytical curve, which correlates the relative concentration of the Fe^2+^ complex to the concentration of the antioxidants.^60^

### 4.11 Oxygen Radical Absorption Capacity (ORAC) Assay

A flat-bottom black microplate was prepared with the following components: 100 μL of a fluorescein solution (400 nM), 50 μL of either RA or NDGA at various concentrations, and 50 μL of 2,2′-azobis­(2-methylpropionamidine)­dihydrochloride (AAPH) at a concentration of 80 mM. The reaction medium used was a 50 mM phosphate buffer at pH 7.0. For the control, the compounds were replaced with 50 μL of the same buffer.^61^ Fluorescence decay was measured at 37 °C, with excitation set to 485 nm and emission detected at 520 nm using a Synergy H1 Hybrid Reader (BioTek, Winooski, VT, USA). Measurements were taken at 5 min intervals over a period of 2 h, with Trolox serving as a reference antioxidant. The area under the curve was calculated, and the results were expressed as μmol of Trolox per μmol of the studied compound.^62^

### 4.12 Statistical Analysis

Data were presented as mean ± standard deviation (SD). Parameters were analyzed by one-way ANOVA followed by Tukey or Dunnett’s multiple-comparison test, as appropriate. For all analyses, *P* values <0.05 were considered statistically significant. Results are presented as mean ± SD of 2–3 independent experiments with 5–8 replicates of cells per treatment condition per assay. All statistical tests were performed using GraphPad Prism 8.0 (GraphPad, San Diego, CA).

## References

[1] Ma Y., Zhu B., Liu X., Yu H., Yong L., Liu X., Shao J., Liu Z. (2015). Inhibition of Oleandrin on the Proliferation
and Invasion of Osteosarcoma Cells in Vitro by Suppressing Wnt/β-Catenin
Signaling Pathway. J. Exp. Clin. Cancer Res..

[2] Nada O. H., Ahmed N. S., Abou
Gabal H. H. (2014). Prognostic Significance of HLA EMR8–5
Immunohistochemically Analyzed Expression in Osteosarcoma. Diagn. Pathol..

[3] Matos A. A., Oliveira F. A., Machado A. C., Saldanha L. L., Tokuhara C. K., Souza L. P., Vilegas W., Dionísio T. J., Santos C., Peres-Buzalaf C., Dokkedal A. L., Oliveira R. (2019). An Extract
from Myracrodruon Urundeuva Inhibits Matrix Mineralization in Human
Osteoblasts. J. Ethnopharmacol..

[4] Kocarnik J. M., Compton K., Dean F. E., Fu W., Gaw B. L., Harvey J. D., Henrikson H. J., Lu D., Pennini A., Xu R., Ababneh E., Abbasi-Kangevari M., Abbastabar H., Abd-Elsalam S. M., Abdoli A., Abedi A., Abidi H., Abolhassani H., Adedeji I. A., Adnani Q. E. S., Advani S. M., Afzal M. S., Aghaali M., Ahinkorah B. O., Ahmad S., Ahmad T., Ahmadi A., Ahmadi S., Ahmed Rashid T., Ahmed Salih Y., Akalu G. T., Aklilu A., Akram T., Akunna C. J., Al Hamad H., Alahdab F., Al-Aly Z., Ali S., Alimohamadi Y., Alipour V., Aljunid S. M., Alkhayyat M., Almasi-Hashiani A., Almasri N. A., Al-Maweri S. A. A., Almustanyir S., Alonso N., Alvis-Guzman N., Amu H., Anbesu E. W., Ancuceanu R., Ansari F., Ansari-Moghaddam A., Antwi M. H., Anvari D., Anyasodor A. E., Aqeel M., Arabloo J., Arab-Zozani M., Aremu O., Ariffin H., Aripov T., Arshad M., Artaman A., Arulappan J., Asemi Z., Asghari
Jafarabadi M., Ashraf T., Atorkey P., Aujayeb A., Ausloos M., Awedew A. F., Ayala Quintanilla B. P., Ayenew T., Azab M. A., Azadnajafabad S., Azari Jafari A., Azarian G., Azzam A. Y., Badiye A. D., Bahadory S., Baig A. A., Baker J. L., Balakrishnan S., Banach M., Bärnighausen T.
W., Barone-Adesi F., Barra F., Barrow A., Behzadifar M., Belgaumi U. I., Bezabhe W. M. M., Bezabih Y. M., Bhagat D. S., Bhagavathula A. S., Bhardwaj N., Bhardwaj P., Bhaskar S., Bhattacharyya K., Bhojaraja V. S., Bibi S., Bijani A., Biondi A., Bisignano C., Bjørge T., Bleyer A., Blyuss O., Bolarinwa O. A., Bolla S. R., Braithwaite D., Brar A., Brenner H., Bustamante-Teixeira M. T., Butt N. S., Butt Z. A., Caetano dos Santos F. L., Cao Y., Carreras G., Catalá-López F., Cembranel F., Cerin E., Cernigliaro A., Chakinala R. C., Chattu S. K., Chattu V. K., Chaturvedi P., Chimed-Ochir O., Cho D. Y., Christopher D. J., Chu D. T., Chung M. T., Conde J., Cortés S., Cortesi P. A., Costa V. M., Cunha A. R., Dadras O., Dagnew A. B., Dahlawi S. M. A., Dai X., Dandona L., Dandona R., Darwesh A. M., das Neves J., De la Hoz F. P., Demis A. B., Denova-Gutiérrez E., Dhamnetiya D., Dhimal M. L., Dhimal M., Dianatinasab M., Diaz D., Djalalinia S., Do H. P., Doaei S., Dorostkar F., dos Santos Figueiredo F. W., Driscoll T. R., Ebrahimi H., Eftekharzadeh S., El Tantawi M., El-Abid H., Elbarazi I., Elhabashy H. R., Elhadi M., El-Jaafary S. I., Eshrati B., Eskandarieh S., Esmaeilzadeh F., Etemadi A., Ezzikouri S., Faisaluddin M., Faraon E. J. A., Fares J., Farzadfar F., Feroze A. H., Ferrero S., Ferro Desideri L., Filip I., Fischer F., Fisher J. L., Foroutan M., Fukumoto T., Gaal P. A., Gad M. M., Gadanya M. A., Gallus S., Gaspar Fonseca M., Getachew Obsa A., Ghafourifard M., Ghashghaee A., Ghith N., Gholamalizadeh M., Gilani S. A., Ginindza T. G., Gizaw A. T. T., Glasbey J. C., Golechha M., Goleij P., Gomez R. S., Gopalani S. V., Gorini G., Goudarzi H., Grosso G., Gubari M. I. M., Guerra M. R., Guha A., Gunasekera D. S., Gupta B., Gupta V. B., Gupta V. K., Gutiérrez R. A., Hafezi-Nejad N., Haider M. R., Haj-Mirzaian A., Halwani R., Hamadeh R. R., Hameed S., Hamidi S., Hanif A., Haque S., Harlianto N. I., Haro J. M., Hasaballah A. I., Hassanipour S., Hay R. J., Hay S. I., Hayat K., Heidari G., Heidari M., Herrera-Serna B. Y., Herteliu C., Hezam K., Holla R., Hossain M. M., Hossain M. B. H., Hosseini M. S., Hosseini M., Hosseinzadeh M., Hostiuc M., Hostiuc S., Househ M., Hsairi M., Huang J., Hugo F. N., Hussain R., Hussein N. R., Hwang B. F., Iavicoli I., Ibitoye S. E., Ida F., Ikuta K. S., Ilesanmi O. S., Ilic I. M., Ilic M. D., Irham L. M., Islam J. Y., Islam R. M., Islam S. M. S., Ismail N. E., Isola G., Iwagami M., Jacob L., Jain V., Jakovljevic M. B., Javaheri T., Jayaram S., Jazayeri S. B., Jha R. P., Jonas J. B., Joo T., Joseph N., Joukar F., Jürisson M., Kabir A., Kahrizi D., Kalankesh L. R., Kalhor R., Kaliyadan F., Kalkonde Y., Kamath A., Kameran Al-Salihi N., Kandel H., Kapoor N., Karch A., Kasa A. S., Katikireddi S. V., Kauppila J. H., Kavetskyy T., Kebede S. A., Keshavarz P., Keykhaei M., Khader Y. S., Khalilov R., Khan G., Khan M. A. B., Khan M. N., Khan M. A. B., Khang Y. H., Khater A. M., Khayamzadeh M., Kim G. R., Kim Y. J., Kisa A., Kisa S., Kissimova-Skarbek K., Kopec J. A., Koteeswaran R., Koul P. A., Koulmane Laxminarayana S. L., Koyanagi A., Kucuk Bicer B., Kugbey N., Kumar G. A., Kumar N., Kumar N., Kurmi O. P., Kutluk T., La Vecchia C., Lami F. H., Landires I., Lauriola P., Lee S. w., Lee S. W. H., Lee W. C., Lee Y. H., Leigh J., Leong E., Li J., Li M. C., Liu X., Loureiro J. A., Lunevicius R., Magdy Abd El Razek M., Majeed A., Makki A., Male S., Malik A. A., Mansournia M. A., Martini S., Masoumi S. Z., Mathur P., McKee M., Mehrotra R., Mendoza W., Menezes R. G., Mengesha E. W., Mesregah M. K., Mestrovic T., Miao Jonasson J., Miazgowski B., Miazgowski T., Michalek I. M., Miller T. R., Mirzaei H., Mirzaei H. R., Misra S., Mithra P., Moghadaszadeh M., Mohammad K. A., Mohammad Y., Mohammadi M., Mohammadi S. M., Mohammadian-Hafshejani A., Mohammed S., Moka N., Mokdad A. H., Molokhia M., Monasta L., Moni M. A., Moosavi M. A., Moradi Y., Moraga P., Morgado-da-Costa J., Morrison S. D., Mosapour A., Mubarik S., Mwanri L., Nagarajan A. J., Nagaraju S. P., Nagata C., Naimzada M. D., Nangia V., Naqvi A. A., Narasimha
Swamy S., Ndejjo R., Nduaguba S. O., Negoi I., Negru S. M., Neupane Kandel S., Nguyen C. T., Nguyen H. L. T., Niazi R. K., Nnaji C. A., Noor N. M., Nuñez-Samudio V., Nzoputam C. I., Oancea B., Ochir C., Odukoya O. O., Ogbo F. A., Olagunju A. T., Olakunde B. O., Omar E., Omar Bali A., Omonisi A. E. E., Ong S., Onwujekwe O. E., Orru H., Ortega-Altamirano D.
V., Otstavnov N., Otstavnov S. S., Owolabi M. O., Pakshir K., Padubidri J. R., Pakshir K., Pana A., Panagiotakos D., Panda-Jonas S., Pardhan S., Park E. C., Park E. K., Pashazadeh Kan F., Patel H. K., Patel J. R., Pati S., Pattanshetty S. M., Paudel U., Pereira D. M., Pereira R. B., Perianayagam A., Pillay J. D., Pirouzpanah S., Pishgar F., Podder I., Postma M. J., Pourjafar H., Prashant A., Preotescu L., Rabiee M., Rabiee N., Radfar A., Radhakrishnan R. A., Radhakrishnan V., Rafiee A., Rahim F., Rahimzadeh S., Rahman M., Rahman M. A., Rahmani A. M., Rajai N., Rajesh A., Rakovac I., Ram P., Ramezanzadeh K., Ranabhat K., Ranasinghe P., Rao C. R., Rao S. J., Rawassizadeh R., Razeghinia M. S., Renzaho A. M. N., Rezaei N., Rezaei N., Rezapour A., Roberts T. J., Rodriguez J. A. B., Rohloff P., Romoli M., Ronfani L., Roshandel G., Rwegerera G. M., Sahebkar A., Sabour S., Saddik B., Saeed U., Sahebkar A., Sahoo H., Salehi S., Salem M. R., Salimzadeh H., Samaei M., Samy A. M., Sanabria J., Sankararaman S., Santric-Milicevic M. M., Sardiwalla Y., Sarveazad A., Sathian B., Sawhney M., Saylan M., Schneider I. J. C., Sekerija M., Seylani A., Shafaat O., Shaghaghi Z., Shaikh M. A., Shamsoddin E., Shannawaz M., Sharma R., Sheikh A., Sheikhbahaei S., Shetty A., Shetty J. K., Shetty P. H., Shibuya K., Shirkoohi R., Shivakumar K. M., Shivarov V., Siabani S., Siddappa Malleshappa S. K., Silva D. A. S., Singh J. A., Sintayehu Y., Skryabin V. Y., Skryabina A. A., Soeberg M. J., Sofi-Mahmudi A., Sotoudeh H., Steiropoulos P., Straif K., Subedi R., Sufiyan M. B., Sultan I., Sultana S., Sur D., Szerencsés V., Szócska M., Tabarés-Seisdedos R., Tabuchi T., Tadbiri H., Taherkhani A., Takahashi K., Talaat I. M., Tan K. K., Tat V. Y., Tedla B. A. A., Tefera Y. G., Tehrani-Banihashemi A., Temsah M. H., Tesfay F. H., Tessema G. A., Thapar R., Thavamani A., Thoguluva Chandrasekar V., Thomas N., Tohidinik H. R., Touvier M., Tovani-Palone M. R., Traini E., Tran B. X., Tran K. B., Tran M. T. N., Tripathy J. P., Tusa B. S., Ullah I., Ullah S., Umapathi K. K., Unnikrishnan B., Upadhyay E., Vacante M., Vaezi M., Valadan
Tahbaz S., Velazquez D. Z., Veroux M., Violante F. S., Vlassov V., Vo B., Volovici V., Vu G. T., Waheed Y., Wamai R. G., Ward P., Wen Y. F., Westerman R., Winkler A. S., Yadav L., Yahyazadeh
Jabbari S. H., Yang L., Yaya S., Yazie T. S. Y., Yeshaw Y., Yonemoto N., Younis M. Z., Yousefi Z., Yu C., Yuce D., Yunusa I., Zadnik V., Zare F., Zastrozhin M. S., Zastrozhina A., Zhang J., Zhong C., Global Burden of Disease 2019 Cancer Collaboration (2022). Cancer Incidence, Mortality,
Years of Life Lost, Years Lived With Disability, and Disability-Adjusted
Life Years for 29 Cancer Groups From 2010 to 2019. JAMA Oncol..

[5] Ritter J., Bielack S. S. (2010). Osteosarcoma. Ann. Oncol..

[6] Li X., Fan S., Pan X., Xiaokaiti Y., Duan J., Shi Y., Pan Y., Tie L., Wang X., Li Y., Li X. (2016). Nordihydroguaiaretic
Acid Impairs Prostate Cancer Cell Migration and Tumor Metastasis by
Suppressing Neuropilin 1. Oncotarget.

[7] Silva, L. R. ; Rodrigues, S. ; Kumar, N. ; Goel, N. ; Singh, K. ; Gonçalves, A. C. Development of Phenolic Acids-Based System as Anticancer Drugs. In Advancement of Phenolic Acids in Drug Discovery; Elsevier, 2024; pp 255–294. 10.1016/B978-0-443-18538-0.00006-8.

[8] Messeha S. S., Zarmouh N. O., Asiri A., Soliman K. F. A. (2020). Rosmarinic Acid-Induced
Apoptosis and Cell Cycle Arrest in Triple-Negative Breast Cancer Cells. Eur. J. Pharmacol..

[9] Villegas C., Cortez N., Ogundele A. V., Burgos V., Pardi P. C., Cabrera-Pardo J. R., Paz C. (2024). Therapeutic Applications of Rosmarinic
Acid in Cancer-Chemotherapy-Associated Resistance and Toxicity. Biomolecules.

[10] Zhao J., Xu L., Jin D., Xin Y., Tian L., Wang T., Zhao D., Wang Z., Wang J. (2022). Rosmarinic Acid and
Related Dietary Supplements: Potential Applications in the Prevention
and Treatment of Cancer. Biomolecules.

[11] de
Vasconcelos D. N., Lima A. N., Philot E. A., Scott A. L., Ferreira Boza I. A., de Souza A. R., Morgon N. H., Ximenes V. F. (2019). Methyl
Divanillate: Redox Properties and Binding Affinity with Albumin of
an Antioxidant and Potential NADPH Oxidase Inhibitor. RSC Adv..

[12] Chipón C., Riffo P., Ojeda L., Salas M., Burgos R. A., Ehrenfeld P., López-Muñoz R., Zambrano A. (2024). Impact of
Nordihydroguaiaretic Acid on Proliferation, Energy Metabolism, and
Chemosensitization in Non-Small-Cell Lung Cancer (NSCLC) Cell Lines. Int. J. Mol. Sci..

[13] Manda G., Rojo A. I., Martínez-Klimova E., Pedraza-Chaverri J., Cuadrado A. (2020). Nordihydroguaiaretic Acid: From Herbal Medicine to
Clinical Development for Cancer and Chronic Diseases. Front. Pharmacol..

[14] Lambert J. D., Zhao D., Meyers R. O., Kuester R. K., Timmermann B. N., Dorr R. T. (2002). Nordihydroguaiaretic
Acid: Hepatotoxicity and Detoxification
in the Mouse. Toxicon.

[15] Moloney J. N., Cotter T. G. (2018). ROS Signalling in
the Biology of Cancer. Semin. Cell Dev. Biol..

[16] Galadari S., Rahman A., Pallichankandy S., Thayyullathil F. (2017). Reactive Oxygen
Species and Cancer Paradox: To Promote or to Suppress?. Free Radic. Biol. Med..

[17] Little A. C., Sulovari A., Danyal K., Heppner D. E., Seward D. J., van der Vliet A. (2017). Paradoxical
Roles of Dual Oxidases in Cancer Biology. Free
Radic. Biol. Med..

[18] Miyata Y., Matsuo T., Sagara Y., Ohba K., Ohyama K., Sakai H. (2017). A Mini-Review of Reactive
Oxygen Species in Urological Cancer: Correlation
with NADPH Oxidases, Angiogenesis, and Apoptosis. Int. J. Mol. Sci..

[19] Paracatu L. C., Quinello Gomes de Faria C. M., Zeraik M. L., Quinello C., Rennó C., Palmeira P., da Fonseca L. M., Ximenes V. F. (2015). Hydrophobicity and
Antioxidant Activity Acting Together
for the Beneficial Health Properties of Nordihydroguaiaretic Acid. Food Funct..

[20] Solak B., Soyer P., Kaya-Tilki E., Öztürk A. A. (2025). Evaluation
of Antimicrobial Properties and Antioxidant Activity of Rosmarinic
Acid Loaded Resomer RG 502 H Based Nanoparticles against Tert-Butyl
Hydroperoxide-Induced Oxidative Stress: A Detailed Formulation, Characterization
and Efficacy Determination Study. J. Drug Delivery
Sci. Technol..

[21] Kale M., Srivastava R. K., Athar M., Banga A. K. (2020). Topical Delivery
of Nordihydroguaretic Acid for Attenuating Cutaneous Damage Caused
by Arsenicals. J. Drug Delivery Sci. Technol..

[22] Avery L. B., Wade J., Wang M., Tam A., King A., Piche-Nicholas N., Kavosi M. S., Penn S., Cirelli D., Kurz J. C., Zhang M., Cunningham O., Jones R., Fennell B. J., McDonnell B., Sakorafas P., Apgar J., Finlay W. J., Lin L., Bloom L., O’Hara D. M. (2018). Establishing *in Vitro in
Vivo* Correlations to Screen Monoclonal Antibodies for Physicochemical
Properties Related to Favorable Human Pharmacokinetics. mAbs.

[23] Mosmann T. (1983). Rapid Colorimetric
Assay for Cellular Growth and Survival: Application to Proliferation
and Cytotoxicity Assays. J. Immunol. Methods.

[24] Carvalho E. P. D., Pessoa A. S., Iano F. G., Ribeiro L., Leme B., Borges L. F., Sanches M. L. R., Ximenes V. F., Oliveira R. C. d. (2024). Antitumor
Effect of Bromo-Naphthoquinone Associated with Tannic Acid in Triple
Negative Breast Cancer Cells. Int. J. Biochem.
Cell Biol..

[25] Kueng W., Silber E., Eppenberger U. (1989). Quantification
of Cells Cultured
on 96-Well Plates. Anal. Biochem..

[26] Pagnan A. L., Pessoa A. S., Tokuhara C. K., Fakhoury V. S., Oliveira G. S. N., Sanches M. L. R., Inacio K. K., Ximenes V. F., Oliveira R. C. (2022). Anti-Tumour Potential and Selectivity
of Caffeic Acid
Phenethyl Ester in Osteosarcoma Cells. Tissue
Cell.

[27] Maurya S., Chaudhary P., Chaudhary A., Singh M., Banoo S., Manna A., Manna P. P. (2026). Design and Synthesis of Protocatechuic
Acid Derivatives for Potential Tumoricidal Effect against Hematological
Malignancies. ACS Omega.

[28] Messeha S. S., Zarmouh N. O., Asiri A., Soliman K. F. A. (2020). Rosmarinic
Acid-Induced
Apoptosis and Cell Cycle Arrest in Triple-Negative Breast Cancer Cells. Eur. J. Pharmacol..

[29] Harrison D. J., Geller D. S., Gill J. D., Lewis V. O., Gorlick R. (2018). Current and
Future Therapeutic Approaches for Osteosarcoma. Expert Rev. Anticancer Ther..

[30] Tobeiha M., Rajabi A., Raisi A., Mohajeri M., Yazdi S. M., Davoodvandi A., Aslanbeigi F., Vaziri M., Hamblin M. R., Mirzaei H. (2021). Potential of Natural
Products in Osteosarcoma Treatment:
Focus on Molecular Mechanisms. Biomed. Pharmacother..

[31] Cai Z., Pan J., He X., Wang W., Zhou Z. (2026). Therapeutic
Potential
of Flavonoids in Osteosarcoma: Molecular Mechanisms and Clinical Implications. Pharmacogn. Mag..

[32] Wang Y., Zhou S., Zhuang Z., An X., Wu W., Jiang B., Zhang C., Manan S., Wang P., Jin Y. (2025). Lignin-Based Therapeutics: Targeting Mitochondrial Pathways in Osteosarcoma. J. Agric. Food Chem..

[33] Pérez-Fons L., GarzÓn M. T., Micol V. (2010). Relationship between
the Antioxidant
Capacity and Effect of Rosemary (Rosmarinus Officinalis L.) Polyphenols
on Membrane Phospholipid Order. J. Agric. Food
Chem..

[34] Alonso M. R., Peralta I., Monti D., Martino R., Anesini C. (2017). Stability
of an Aqueous Extract of *Larrea Divaricata* Cav. during
a Simulated Digestion Process. Phytother. Res..

[35] Zhang H., Tsao R. (2016). Dietary Polyphenols,
Oxidative Stress and Antioxidant and Anti-Inflammatory
Effects. Curr. Opin. Food Sci..

[36] Lin C., Xiao J., Xi Y., Zhang X., Zhong Q., Zheng H., Cao Y., Chen Y. (2019). Rosmarinic Acid Improved
Antioxidant Properties and Healthspan via the IIS and MAPK Pathways
in *Caenorhabditis Elegans*. BioFactors.

[37] Lin S.-K., Kok S.-H., Kuo M. Y.-P., Lee M.-S., Wang C.-C., Lan W.-H., Hsiao M., Goldring S. R., Hong C.-Y. (2003). Nitric
Oxide Promotes Infectious Bone Resorption by Enhancing Cytokine-Stimulated
Interstitial Collagenase Synthesis in Osteoblasts. J. Bone Miner. Res..

[38] Orhaner C., Orhaner A., Tuncer M. C., Özdemir I. ˙. (2026). Effects
of Rosmarinic Acid and Doxorubicin Combination in Breast Cancer Cells. Biology.

[39] Panieri E., Santoro M. M. (2016). ROS Homeostasis
and Metabolism: A Dangerous Liaison
in Cancer Cells. Cell Death Dis..

[40] Trachootham D., Alexandre J., Huang P. (2009). Targeting Cancer Cells
by ROS-Mediated
Mechanisms: A Radical Therapeutic Approach?. Nat. Rev. Drug Discovery.

[41] Sosa V., Moliné T., Somoza R., Paciucci R., Kondoh H., Lleonart M. E. (2013). Oxidative Stress and Cancer: An Overview. Ageing Res. Rev..

[42] Nguyen C., Pandey S. (2019). Exploiting Mitochondrial Vulnerabilities
to Trigger
Apoptosis Selectively in Cancer Cells. Cancers.

[43] Biswal S. S. (2000). Glutathione
Oxidation and Mitochondrial Depolarization as Mechanisms of Nordihydroguaiaretic
Acid-Induced Apoptosis in Lipoxygenase-Deficient FL5.12 Cells. Toxicol. Sci..

[44] Mayor R., Etienne-Manneville S. (2016). The Front and Rear of Collective Cell Migration. Nat. Rev. Mol. Cell Biol..

[45] Souza F. da C., Ferreira M. T., Colquhoun A. (2020). Influence of Lipoxygenase Inhibition
on Glioblastoma Cell Biology. Int. J. Mol. Sci..

[46] Manciu F. S., Guerrero J., Bennet K. E., Chang S.-Y., Rahman M., Martinez Lopez L. V., Chantigian S., Castellanos M., Manciu M. (2022). Assessing Nordihydroguaiaretic Acid
Therapeutic Effect
for Glioblastoma Multiforme. Sensors.

[47] Ma Z., Yang J., Yang Y., Wang X., Chen G., Shi A., Lu Y., Jia S., Kang X., Lu L. (2020). Rosmarinic
Acid Exerts an Anticancer Effect on Osteosarcoma Cells by Inhibiting
DJ-1 via Regulation of the PTEN-PI3K-Akt Signaling Pathway. Phytomedicine.

[48] Lee C. M., Lee J., Nam M. J., Park S.-H. (2018). Indole-3-Carbinol Induces Apoptosis
in Human Osteosarcoma MG-63 and U2OS Cells. BioMed Res. Int..

[49] Macena J. C., Renzi D. F., Grigoletto D. F. (2022). Chemical
and Biological Properties
of Nordihydroguaiaretic Acid. Braz. J. Pharm.
Sci..

[50] Lambert J. D., Zhao D., Meyers R. O., Kuester R. K., Timmermann B. N., Dorr R. T. (2002). Nordihydroguaiaretic
Acid: Hepatotoxicity and Detoxification
in the Mouse. Toxicon.

[51] Quarles L. D., Yohay D. A., Lever L. W., Caton R., Wenstrup R. J. (1992). Distinct
Proliferative and Differentiated Stages of Murine MC3T3-E1 Cells in
Culture: An in Vitro Model of Osteoblast Development. J. Bone Miner. Res..

[52] Inacio K. K., Pessoa A. d. S., Tokuhara C. K., Pagnan A. L., Sanches M. L. R., Fakhoury V. S., Oliveira G. S. N. d., Oliveira F. A. d., Ximenes V. F., Oliveira R. C. d. (2024). Menadione and Protocatechuic Acid: A Drug Combination
with Antitumor Effects in Murine Osteosarcoma Cells. Arch. Biochem. Biophys..

[53] Fakhoury V. S., PessoaTokuharaPagnan A. d. C. K. A. L., Oliveira G. S. N. d., Liessa M. R. S., Inacio K. K., Melo F. P. d. S. R. d., Dokkedal A. L., Oliveira R. C. d., Saldanha L. L., Oliveira R. C. d., Saldanha L. (2022). Evaluation of *Myrcia Bella* in Murine
Osteosarcoma Cells: Effect of the Extract and Enriched Fractions of
Tannins and Flavonoids. Nat. Prod. Res..

[54] Feoktistova M., Geserick P., Leverkus M. (2016). Crystal Violet
Assay for Determining
Viability of Cultured Cells. Cold Spring Harb.
Protoc..

[55] Salomão P. M.
A., Oliveira F. A. d., Rodrigues P. D., Al-Ahj L. P., Gasque K. C. d. S., Jeggle P., Buzalaf M. A. R., Oliveira R. C. d., Edwardson J. M., Magalhães A. C. (2017). The Cytotoxic Effect of TiF4 and NaF on Fibroblasts
Is Influenced by the Experimental Model, Fluoride Concentration and
Exposure Time. PLoS One.

[56] Inacio K. K., Pessoa A. d. S., Tokuhara C. K., Pagnan A. L., Sanches M. L. R., Fakhoury V. S., Oliveira G. S. N. d., Oliveira F. A. d., Ximenes V. F., Oliveira R. C. d. (2024). Menadione
and Protocatechuic Acid: A Drug Combination
with Antitumor Effects in Murine Osteosarcoma Cells. Arch. Biochem. Biophys..

[57] Andrade
Carvalho A., da Costa P. M., Da Silva Souza L. G., Lemos T. L. G., Alves A. P. N. N., Pessoa C., de Moraes M. O. (2013). Inhibition
of Metastatic Potential of B16-F10 Melanoma Cell Line in Vivo and
in Vitro by Biflorin. Life Sci..

[58] Pessoa A. d. S., Tokuhara C. K., Fakhoury V. S., Pagnan A. L., Oliveira G. S. N. d., Sanches M. L. R., Inacio K. K., Costa B. C., Ximenes V. F., Oliveira R. C. d. (2022). The Dimerization
of Methyl Vanillate
Improves Its Effect against Breast Cancer Cells via Pro-Oxidant Effect. Chem.-Biol. Interact..

[59] Ximenes V. F., Lopes M. G., Petrônio M. S., Regasini L. O., Siqueira
Silva D. H., da Fonseca L. M. (2010). Inhibitory Effect of Gallic Acid
and Its Esters on 2,2′-Azobis­(2-Amidinopropane)­Hydrochloride
(AAPH)-Induced Hemolysis and Depletion of Intracellular Glutathione
in Erythrocytes. J. Agric. Food Chem..

[60] de
Vasconcelos D. N., Lima A. N., Philot E. A., Scott A. L., Ferreira Boza I. A., de Souza A. R., Morgon N. H., Ximenes V. F. (2019). Methyl
Divanillate: Redox Properties and Binding Affinity with Albumin of
an Antioxidant and Potential NADPH Oxidase Inhibitor. RSC Adv..

[61] Zazeri G., Povinelli A. P. R., Bertozo L. d. C., Jones A. M., Ximenes V. F. (2024). The Role
of Medium Polarity in the Efficiency of Albumin Binding with Hydrophobic
Ligands: Experimental Studies and a Molecular Dynamics Investigation. Int. J. Mol. Sci..

[62] Ou B., Hampsch-Woodill M., Prior R. L. (2001). Development and Validation of an
Improved Oxygen Radical Absorbance Capacity Assay Using Fluorescein
as the Fluorescent Probe. J. Agric. Food Chem..

